# Robotic resection for splenic artery aneurysm associated with neurofibromatosis type 1: a case report

**DOI:** 10.1186/s13256-024-04440-3

**Published:** 2024-03-14

**Authors:** Akihiko Ueda, Kenta Saito, Hiromichi Murase, Tomokatsu Kato, Hiroyuki Imafuji, Mamoru Morimoto, Ryo Ogawa, Hiroki Takahashi, Yoichi Matsuo, Shuji Takiguchi

**Affiliations:** Department of Gastroenterological Surgery, Nagoya City University Graduate School of Medical Sciences, 1 Kawasumi, Mizuho-Cho, Mizuho-Ku, Nagoya, Aichi 4678601 Japan

**Keywords:** Neurofibromatosis type 1, Splenic artery aneurysm, Robotic surgery, Von Recklinghausen’s disease, Case report

## Abstract

**Background:**

Neurofibromatosis type 1 is an autosomal-dominant disease characterized by café-au-lait spots and neurofibromas, as well as various other symptoms in the bones, eyes, and nervous system. Due to its connection with vascular fragility, neurofibromatosis type 1 has been reported to be associated with vascular lesions, such as aneurysms. However, there have been few reports of abdominal visceral aneurysms associated with neurofibromatosis type 1. Furthermore, there have been no reports of robotic treatment of aneurysms associated with neurofibromatosis type 1. In this report, we describe the case of a patient with neurofibromatosis type 1 with a splenic artery aneurysm who was successfully treated with robotic surgery.

**Case presentation:**

This report describes a 41-year-old Asian woman with a history of neurofibromatosis type 1 who was referred to our hospital for evaluation of a 28 mm splenic artery aneurysm observed on abdominal ultrasound. The aneurysm was in the splenic hilum, and transcatheter arterial embolization was attempted; however, this was difficult due to the tortuosity of the splenic artery. Thus, we suggested minimally invasive robotic surgery for treatment and resection of the splenic artery aneurysm with preservation of the spleen. The postoperative course was uneventful, and the patient was discharged on the eighth day after surgery. At 1 year of follow-up, the patient was doing well, with no evidence of recurrence.

**Conclusion:**

We encountered a rare case of splenic artery aneurysm in a patient with neurofibromatosis type 1 who was successfully treated with robotic surgery. There is no consensus on treatment modalities for neurofibromatosis-related aneurysms, and endovascular treatment is considered safe and effective; however, surgery remains an important treatment modality. Especially in patients with stable hemodynamic status, robotic surgery may be considered as definitive treatment. To our knowledge, this is the first successfully treated case of a splenic artery aneurysm in a patient with neurofibromatosis type 1.

## Background

Neurofibromatosis type 1 (NF1), also known as von Recklinghausen’s disease, is an autosomal-dominant disease that causes various manifestations such as multiple flat, light-brown patches of skin pigment (café-au-lait spots), freckling of skinfolds, visible neurofibromas under the skin, and small nodules of the iris (Lisch nodules) [1, 2]. NF1 occurs in 1 in 3000–4000 people worldwide. Due to its connection to vascular fragility, NF1 has been reported to be associated with vasculopathy, with vascular lesions reported to complicate 3–9% of cases [3]. The spectrum of vasculopathy in NF1 includes aneurysms, stenosis, and arteriovenous malformations of medium- and large-sized vessels. Although there have been some reports of abdominal visceral arteries such as the liver artery, superior mesenteric artery (SMA), and inferior mesenteric artery (IMA) [4–6], there have been no reports of splenic artery aneurysms, and this is believed to be the first reported aneurysm of this type worldwide.

Aneurysms are often asymptomatic and may be found incidentally on imaging studies; however, they have the potential to be fatal due to life-threatening bleeding from ruptured aneurysms [7, 8]. Careful medical management, such as avoiding hypertension, is first necessary to avoid aneurysm rupture. Surgical intervention is guided by the age and comorbidities of the patient as well as the site and type of aneurysm. Open aneurysmectomy or endovascular treatment is often the treatment of choice, but there have been no reports of robotic treatment of aneurysms associated with NF1. In this report, we describe the case of a patient with NF1 with a splenic aneurysm who was successfully treated with robotic surgery.

## Case presentation

**Fig. 1:**
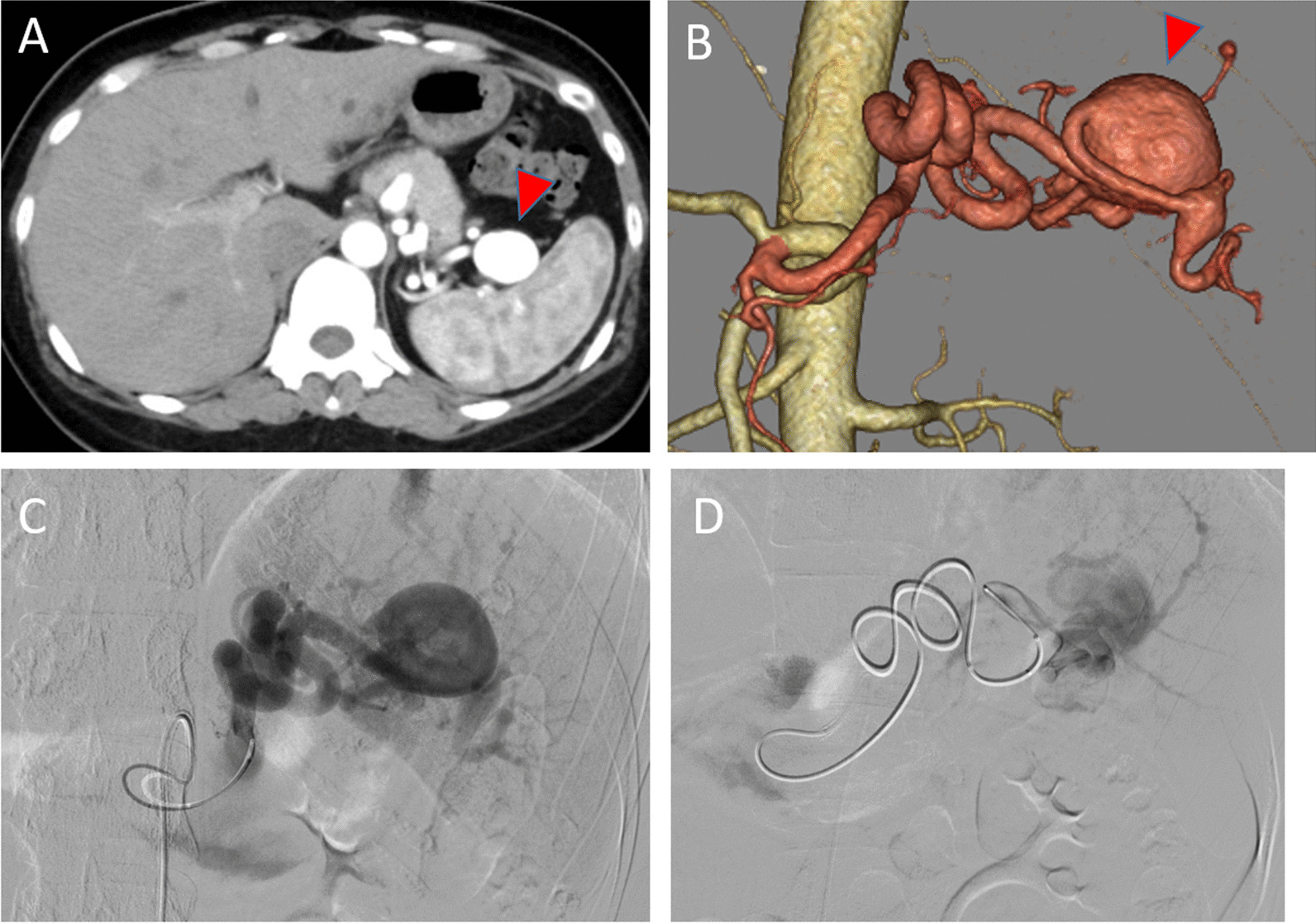
**A**, **B** Contrast-enhanced abdominal computed tomography showed a 28-mm splenic artery aneurysm at the splenic hilum (arrowhead). **C** Angiography showed a splenic artery aneurysm. **D** Transcatheter arterial embolization was attempted; however, this was difficult due to tortuosity of the splenic artery

**Fig. 2:**
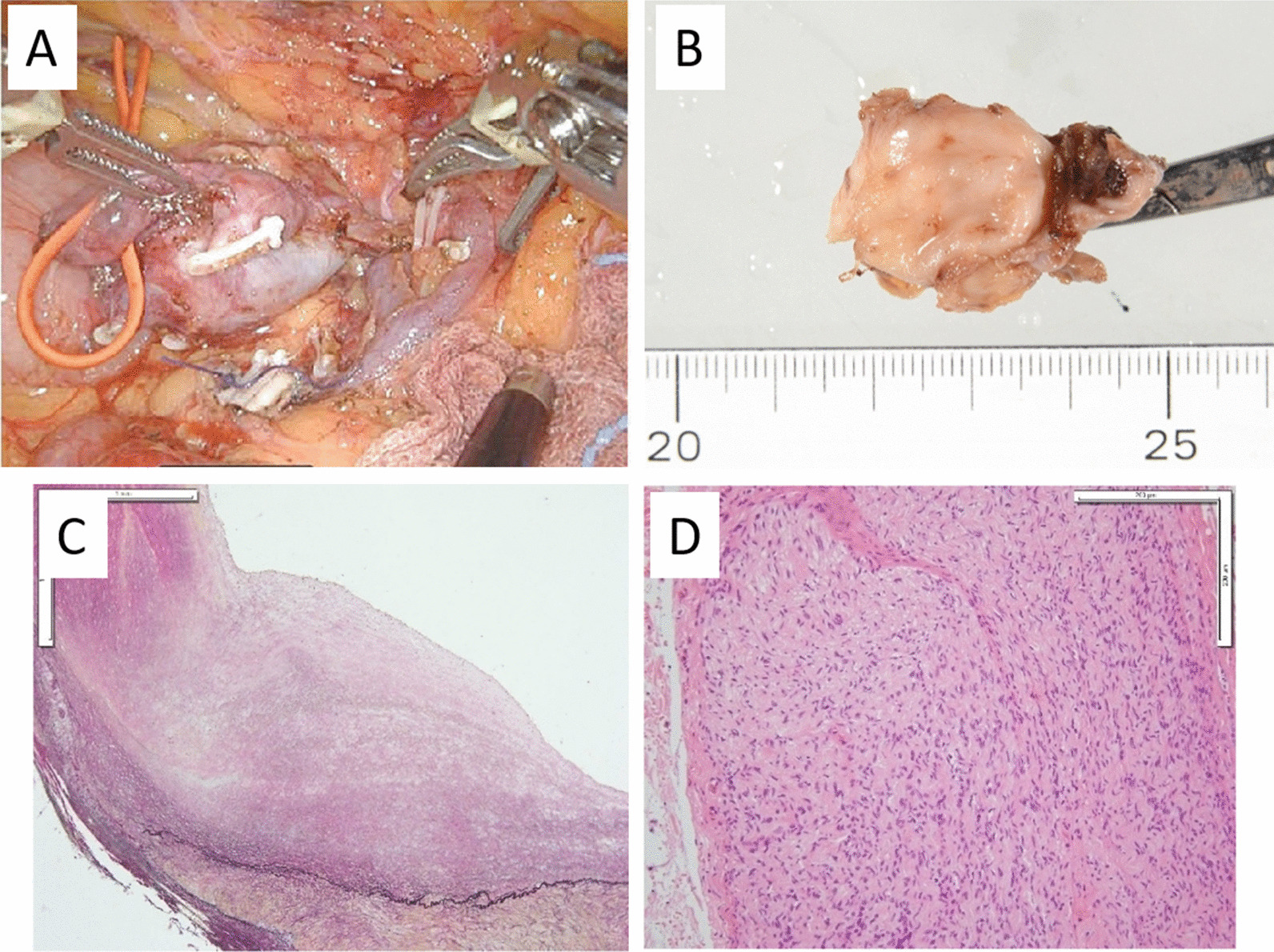
**A** Robotic aneurysmectomy was performed, and the spleen could be preserved. **B** Resected specimen of splenic artery aneurysm. **C** A histopathological examination showed severe intimal thickening of the aneurysm, and the internal elastic lamina over half of the circumference had disappeared. **D** Neurofibroma attached to the adventitia were observed

A 41-year-old Asian woman was referred to our hospital for evaluation of a splenic artery aneurysm noted on abdominal ultrasound. The patient had no subjective symptoms. The patient’s clinical history included neurofibromatosis type 1, Noonan syndrome, peptic gastric ulcer, endometriosis, and ventricular septal defects. At the age of 27 years, the patient underwent excision of a cutaneous nodule on the right thigh, the pathological diagnosis of which was plexiform neurofibroma. The patient had no relevant family history of vasculopathy, and no psychosocial history. She had no history of regular medications. The patient had no history of smoking or alcohol consumption. The patient was 151 cm tall and weighed 60 kg. On the initial medical examination, there were no relevant physical or neurological findings, her blood pressure was 125/77 mmHg, and her pulse rate was 85 beats per minute. The patient’s laboratory data were within normal limits. Contrast-enhanced computed tomography (CT) showed a 28-mm aneurysm in the splenic hilum (Fig. 1A, B). Angiography revealed similar findings (Fig. 1C). She was asymptomatic, but because her splenic artery aneurysm was > 20 mm in diameter, it had the potential to rupture, causing life-threatening complications. Therefore, we determined that it was indicated for treatment. Transcatheter arterial embolization (TAE) was attempted, but it was difficult due to the tortuosity of the splenic artery (Fig. 1D). Therefore, we suggested surgery, especially minimally invasive procedures, such as laparoscopic and robotic techniques. The patient underwent robotic resection of the splenic artery aneurysm with preservation of the spleen (Fig. 2A). The postoperative course was uneventful, and the patient was discharged on the eighth day after surgery. At 1 year of follow-up, the patient was doing well, with no evidence of recurrence.

### Pathological findings

The gross examination revealed that the aneurysm was approximately 20 mm in size (Fig. 2B). A histopathological examination showed severe intimal thickening of the aneurysm, with the internal elastic lamina of over half of the circumference having disappeared (Fig. 2C). Neurofibromas attached to adventitia were also observed (Fig. 2D). The pathological diagnosis was a splenic artery aneurysm associated with neurofibromatosis.

## Discussion

Neurofibromatosis type 1 (NF1), also known as von Recklinghausen’s disease, is an autosomal dominant disease that causes various manifestations such as multiple flat, light-brown patches of skin pigment (café-au-lait spots), freckling of the skinfolds, visible neurofibromas under the skin, and small nodules of the iris (Lisch nodules) [1, 2]. NF1 occurs in 1 in 3000–4000 people worldwide. Due to vascular fragility, NF1 has been reported to be associated with vascular lesions, such as stenosis, occlusion, aneurysm, arteriovenous malformation, and arteriovenous fistula, with vascular complications reported in 3–9% of cases [3]. The first site where vascular lesions are most likely to occur is the renal artery, followed by the carotid and cerebral arteries, and the subclavian and vertebral arteries [9]. Stenosis of the renal arteries results in renal vascular hypertension [10]. The vascular lesions are often asymptomatic; however, they have the potential to be fatal due to massive bleeding from ruptured aneurysms [7, 8] and are the second leading cause of death after soft tissue malignancies in NF1 patients [9]. Several hypotheses have been proposed to explain the mechanism by which NF1 causes aneurysms [11, 12]: (1) weakening of the vessel wall due to direct infiltration of the neurofibroma into the intima; (2) weakening of the artery due to ischemia caused by compression of the nutrient vessels by the neurofibroma; and (3) weakening of the endoelastic plate due to flattening of the tunica media support cells by proliferation of spindle-shaped cells in the intima.

Treatment options for aneurysms include surgical resection of the aneurysm with or without revascularization and endovascular procedures such as coil embolization and stent grafting [13]. Although there is no consensus on treatment modalities, a systematic review of endovascular treatment of neurofibromatosis-related aneurysms reported that endovascular treatment is safe and effective, regardless of age or hemodynamic status [14]. However, endovascular treatment is difficult to perform when there is vascular tortuosity or when there is a risk of impaired blood flow to the peripheral organs. In splenic artery aneurysms, endovascular treatment is often the first choice because of its superior perioperative mortality and short-term results; however, surgery is performed when endovascular treatment is considered difficult [15, 16].

In the general population, splenic aneurysms are the most common type of abdominal visceral aneurysms, accounting for approximately 60% [17], with an estimated prevalence of 0.8% [18]. In contrast, in a report examining 40 aneurysm lesions associated with NF1, there were only two cases involving the liver artery, one case involving the gastroduodenal artery, and one case involving the pancreaticoduodenal artery, indicating that abdominal visceral aneurysms are rare [9]. There have been several reports of abdominal visceral aneurysms associated with NF1, including liver, superior mesenteric, inferior mesenteric, celiac, gastroduodenal, and superior rectal arteries [4–6, 9, 19–34] (Table 1). However, to our knowledge, there are no reports of splenic aneurysms associated with NF1 in the relevant English literature, and we believe that this is the first report of such an aneurysm. The reason why which splenic aneurysms are more frequent in the general population but less frequent in the NF1 population is unclear, but the results are interesting.

**Table 1:** Reported cases of visceral artery aneurysm associated with neurofibromatosis type 1

Author, year	Case; M or F	Location	Symptoms; rupture	Size (mm)	Treatment
Huffman, 1996	44; M	SMA	Abdominal pain; yes	60	Laparotomy
Hassen-Khodja, 1997	55; F	CHA	Right lower quadrant pain; yes	40	Endovascular treatment (coil embolization)
Serleth, 1998	20; F	PDA	Abdominal pain; yes	N/A	Laparotomy
Sacar, 2006	20; M	IMA	Asymptomatic; no	25	Laparotomy
Oderich, 2007	12; F	HA	Pain; yes	N/A	Endovascular treatment (coil embolization)
Rao, 2007	48; M	RHA	Central chest pain; yes	N/A	Endovascular treatment (coil embolization)
Mendonça, 2010	31; F	SMA	Abdominal pain; yes	25	Endovascular treatment (stent)
Kerger, 2012	61; F	IMA	Abdominal pain; yes	25	Laparotomy
Makino, 2013	39; M	SRA	Loss of consciousness; yes	N/A	Endovascular treatment (coil embolization)
Morris, 2013	16; M	RHA	Jaundice; no	50	Endovascular treatment (coil embolization)
Im, 2015	43; M	GDA	Hematemesis; yes	N/A	Endovascular treatment (coil embolization)
Yow, 2015	55; F	SRA	Abdominal pain; yes	N/A	Laparotomy after failed endovascular treatment
Lim, 2016	51; F	LHA	Epigastric pain; yes	N/A	Endovascular treatment (coil embolization)
Sheth, 2018	64; F	IMA	Lower abdominal pain; yes	N/A	Laparotomy
Drucker, 2019	18; F	PDA	Abdominal pain; no	N/A	Endovascular treatment (stent)
Moro, 2019	67; F	LCA	Shock; yes	N/A	Laparotomy
Fukushima, 2020	55; F	PDA/SMA	Abdominal pain; yes	N/A	Endovascular treatment (coil embolization)
Nemoto, 2022	52; F	SRA	Back pain; yes	30	Endovascular treatment (coil embolization)
Takata, 2022	42; F	CA	Shock; yes	14	Hybrid treatment (stent, laparotomy)
Rajahram, 2023	60s; M	CA	Epigastric pain; yes	N/A	Laparotomy
Present case	41; F	SA	Asymptomatic; no	28	Robotic surgery

*CA* celiac artery, *CHA* common hepatic artery, *GDA* gastroduodenal artery, *HA* hepatic artery, *IMA* inferior mesenteric artery, *LCA* left colic artery, *LHA* left hepatic artery, *PDA* pancreaticoduodenal artery, *RHA* right hepatic artery, *SA* splenic artery, *SMA* superior mesenteric artery, *SRA* superior rectal artery

In recent years, remarkable progress has been made in minimally invasive surgery, especially robotic surgery, and its indications are expanding. Robotic surgery is considered to enable more precise manipulation due to its features, such as a wide range of motion with multiple joints, a high-magnification 3D field of view, and sufficient traction without shaking. Rompianesi *et al.* compared robotic and laparoscopic pancreaticoduodenectomy with spleen preservation and reported a higher rate of spleen preservation with robotic surgery [35]. A systematic review of laparoscopic and robotic surgeries for splenic aneurysms reported that 3.6% of laparoscopic patients underwent distal pancreatectomy, while no patients underwent pancreatectomy in robotic surgery, and 29.8% of laparoscopic patients underwent splenectomy, while 9.1% underwent splenectomy in robotic surgery [36]. In splenic artery aneurysmectomy, robotic surgery may contribute to preservation of the pancreas and spleen because of its dexterity.

The present case was asymptomatic, and the aneurysm was discovered incidentally. Pathological findings showed a neurofibroma infiltrating the adventitia, and the ischemic effect due to pressure drainage was suspected to be the mechanism of pathogenesis. The first step of treatment would have been a minimally invasive endovascular embolization procedure; however, the vascular tortuosity was so severe that surgical treatment was necessary. As the patient had a stable hemodynamic status, we presented robotic surgical treatment, which is considered to be one of the less invasive techniques in surgery. As a result, the patient was able to benefit from minimally invasive surgery, with preservation of the pancreas and spleen and early discharge without perioperative complications.

## Conclusions

We encountered a rare case of splenic artery aneurysm in a patient with NF1 who was successfully treated by robotic surgery. In the present case, robotic aneurysmectomy was accomplished with preservation of the pancreas and spleen, suggesting that robotic surgery may be useful for preserving the pancreas and spleen in splenic aneurysmectomy. The present case suggests that robotic surgery can be performed safely even in NF1 cases, which are considered to have vascular fragility. Especially in patients with stable hemodynamic status, robotic surgery may be considered as definitive treatment. To our knowledge, this is the first successfully treated case of a splenic artery aneurysm in a patient with NF1.

## Data Availability

The datasets supporting the conclusions of this article are included within the article.

## Abbreviations

CT: Computed tomography
IMA: Inferior mesenteric artery
NF1: Neurofibromatosis type 1
SMA: Superior mesenteric artery
TAE: Transcatheter arterial embolization
